# Partial aperture imaging system based on sparse point spread holograms and nonlinear cross-correlations

**DOI:** 10.1038/s41598-020-77912-3

**Published:** 2020-12-15

**Authors:** Angika Bulbul, Joseph Rosen

**Affiliations:** School of Electrical and Computer Engineering, Ben-Gurion University of the Negev, P.O. Box 653, 8410501 Beer-Sheva, Israel

**Keywords:** Applied optics, Optical techniques, Optics and photonics

## Abstract

Partial aperture imaging system (PAIS) is a recently developed concept in which the traditional disc-shaped aperture is replaced by an aperture with a much smaller area and yet its imaging capabilities are comparable to the full aperture systems. Recently PAIS was demonstrated as an indirect incoherent digital three-dimensional imaging technique. Later it was successfully implemented in the study of the synthetic marginal aperture with revolving telescopes (SMART) to provide superresolution with subaperture area that was less than one percent of the area of the full synthetic disc-shaped aperture. In the study of SMART, the concept of PAIS was tested by placing eight coded phase reflectors along the boundary of the full synthetic aperture. In the current study, various improvements of PAIS are tested and its performance is compared with the other equivalent systems. Among the structural changes, we test ring-shaped eight coded phase subapertures with the same area as of the previous circular subapertures, distributed along the boundary of the full disc-shaped aperture. Another change in the current system is the use of coded phase mask with a point response of a sparse dot pattern. The third change is in the reconstruction process in which a nonlinear correlation with optimal parameters is implemented. With the improved image quality, the modified-PAIS can save weight and cost of imaging devices in general and of space telescopes in particular. Experimental results with reflective objects show that the concept of coded aperture extends the limits of classical imaging.

## Introduction

Shape and size of optical apertures dictate the image resolution, depth of field and other image qualities in the entire optical imaging systems, such as microscopes, telescopes and cameras. Properties like contrast, sharpness, noise and artifacts are also affected by the exact shape of the aperture, hence an optimized shape of aperture can be useful in optical instrumentation. For example, the axial resolution can be optimized by placing two centrosymmetric D-shaped apertures at an optimal separation^1^. Another example is a ring-shaped aperture of 30 m diameter studied for implementing in a light-weight space telescope^2^. Not only the shape but the space-time distribution of apertures can also contribute to the resolution enhancement. Interferometric telescopes equipped with two primary subapertures are typical examples for unusual optical space-time-distributed apertures^3^.

Another family of apertures is the group of coded apertures in which not only the aperture shape but also the transparency distribution is considered. Coded apertures, in general, are sorted to coded amplitude masks (CAMs) and coded phase masks (CPMs). An example of the CAM is the binary pattern displayed on the camera aperture to improve certain features of the recoded image by digital post-processing^4^. The history of CAMs is rooted in the sixties with the works of Dicke and Ables in the field of X-ray astronomy. In these works, the entrance of a pinhole camera was replaced by several randomly distributed pinholes to improve the signal-to-noise ratio (SNR) of X-ray astronomical images^5,6^. CAMs have been also used in medical imaging to perform tomography for X-ray imaging^7^. In compressed sensing, CAMs are used for high resolution compressed imaging^8^, video reconstructions^9^ and as a dynamic lensless camera^10^.

The concept of CPM is more recent compared to CAM and has been studied in the frame of coded aperture correlation holography (COACH)^11–15^. COACH is an inline incoherent digital holographic technique which was proposed as a generalization of the special case of Fresnel incoherent correlation holography^16–18^. Interferenceless-coded aperture correlation holography (I-COACH), suggested about a year after the appearance of COACH, eliminates the drawbacks related to two-beam interference^12^. Because beam-splitting and the interference are avoided, I-COACH is more power-efficient and noise-immune than COACH.

The concepts of apertures with unusual shapes and I-COACH are combined in the study of the partial aperture imaging system (PAIS)^19^. PAIS is an incoherent digital holographic technique based on the I-COACH, in which only part of the full disc-shaped aperture is open for light transmission. In the first work of PAIS, an annular coded aperture along the boundary of the full aperture was studied^19^. With an annular area of 1.4% of the full aperture, the resulting images have had an image resolution similar to that of a full aperture imaging system with the same numerical aperture (*NA*). The resolution of an imaging system depends on the wavelength of light *λ* and the *NA* of the system. The lateral minimal resolved size is $0.61\lambda {/}NA$ and the axial minimal resolved size is $\left( {2\lambda {/}NA^{2} } \right),$ where *NA* = *n*sin*θ*, *n* is the refractive index of the space between the target and the imaging system and *θ* is the angle between the marginal ray and the *z*-axis^20^. Thus, the size of the aperture determines the resolution and light-gathering power. To increase the effective *NA,* synthetic aperture imaging is commonly used in radio^21^, submillimeter^22^, infrared^23^ and visible wavelengths^24^. Recently, the concept of PAIS has been extended for the method of the synthetic marginal aperture with revolving telescopes (SMART)^25^. To imitate eight revolving satellites, eight circular subapertures, equally distributed along the boundary of a full synthetic aperture, replaced the annular aperture. Each moment, only two subapertures were employed simultaneously and the final image was the sum of the entire accumulated images of subaperture pairs.

**Figure 1:**
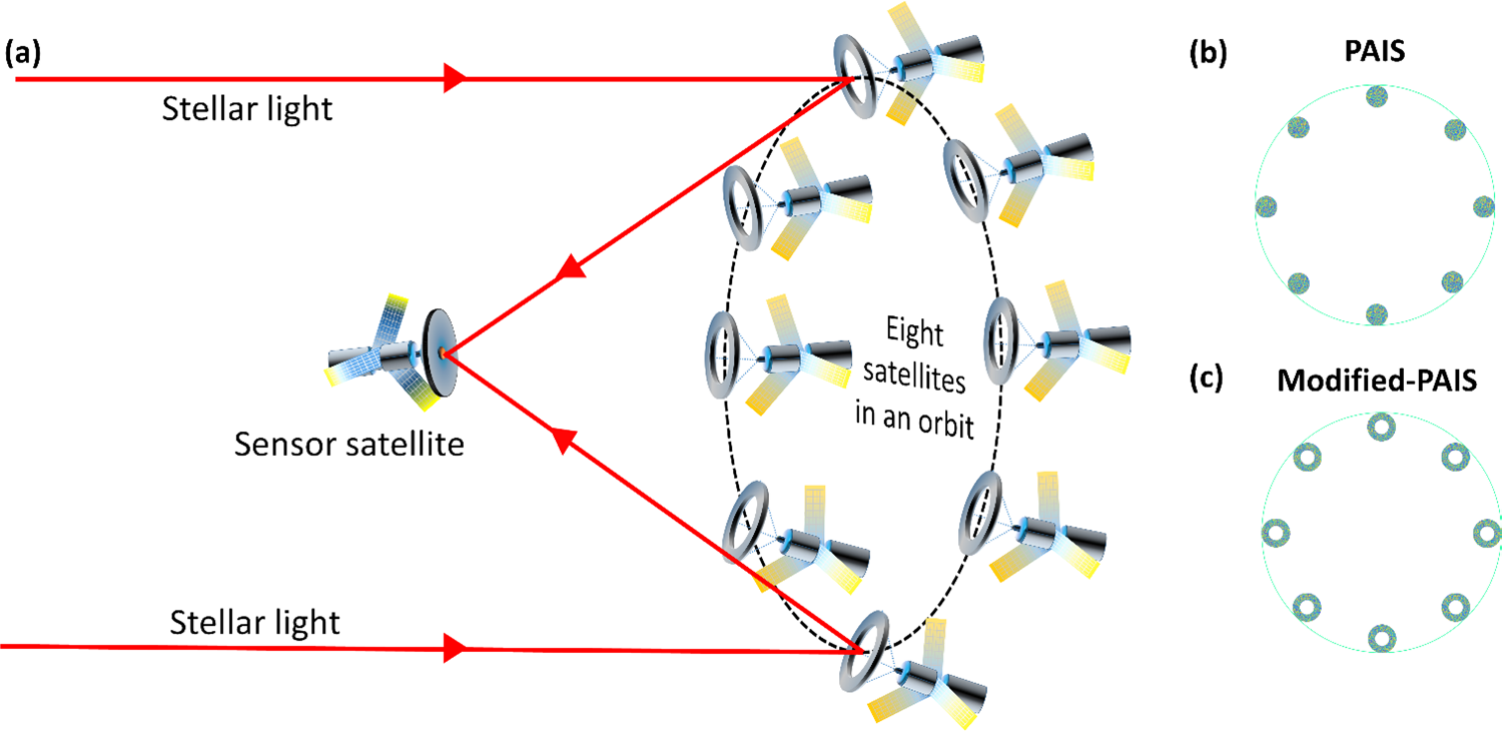
(**a**) A scheme of implementing M-PAIS for a possible future space telescope, (**b**) and (**c**) CPMs of PAIS and M-PAIS, respectively.

In this study, we demonstrate a modified partial aperture imaging system (M-PAIS), in purpose to improve the performance of the original PAIS. In M-PAIS the coded subapertures are synthesized in ring-shaped subapertures, with the same area as of circular-shaped subapertures, to further increase the resolution of the PAIS system without using more resources. In other words, the number of pixels needed to synthesize pseudorandom CPMs of PAIS and M-PAIS is the same. In the following, we show that the configuration of M-PAIS significantly uses less resources (less aperture area) than a full aperture imager without compromising on the image resolution. Figure 1a demonstrates schematically the M-PAIS operation as a possible future space telescope. The pseudorandom CPMs of PAIS and M-PAIS are shown in Fig. 1b and 1c, respectively. M-PAIS is a far-field imaging system and assuming the range to the observed target cannot be changed, only extending the aperture size can increase the *NA* and thus it can improve the image resolution.

Besides the new shape of the sub-apertures in M-PAIS, we introduce herein three additional modifications in comparison to the original PAIS^19^. First, the entire experiment is performed with reflective targets, making the setup closer to real-world applications. Second, based on the study of sparse I-COACH^26^, we improve the SNR by use of an imager with sparse point response. Third, the reconstructed images are further improved by the use of nonlinear reconstruction (NLR)^27,28^. NLR is also used as a way to achieve real-time imaging in PAIS systems with a single camera shot. Although NLR with one camera shot is sufficient for image retrieval, using NLR with two camera shots further reduces the noise.

The manuscript contains four sections. The second section presents the theory and the principles of M-PAIS. In the third section, experiments are discussed and the results are analyzed. The final section summarizes this study.

## Methodology

**Figure 2:**
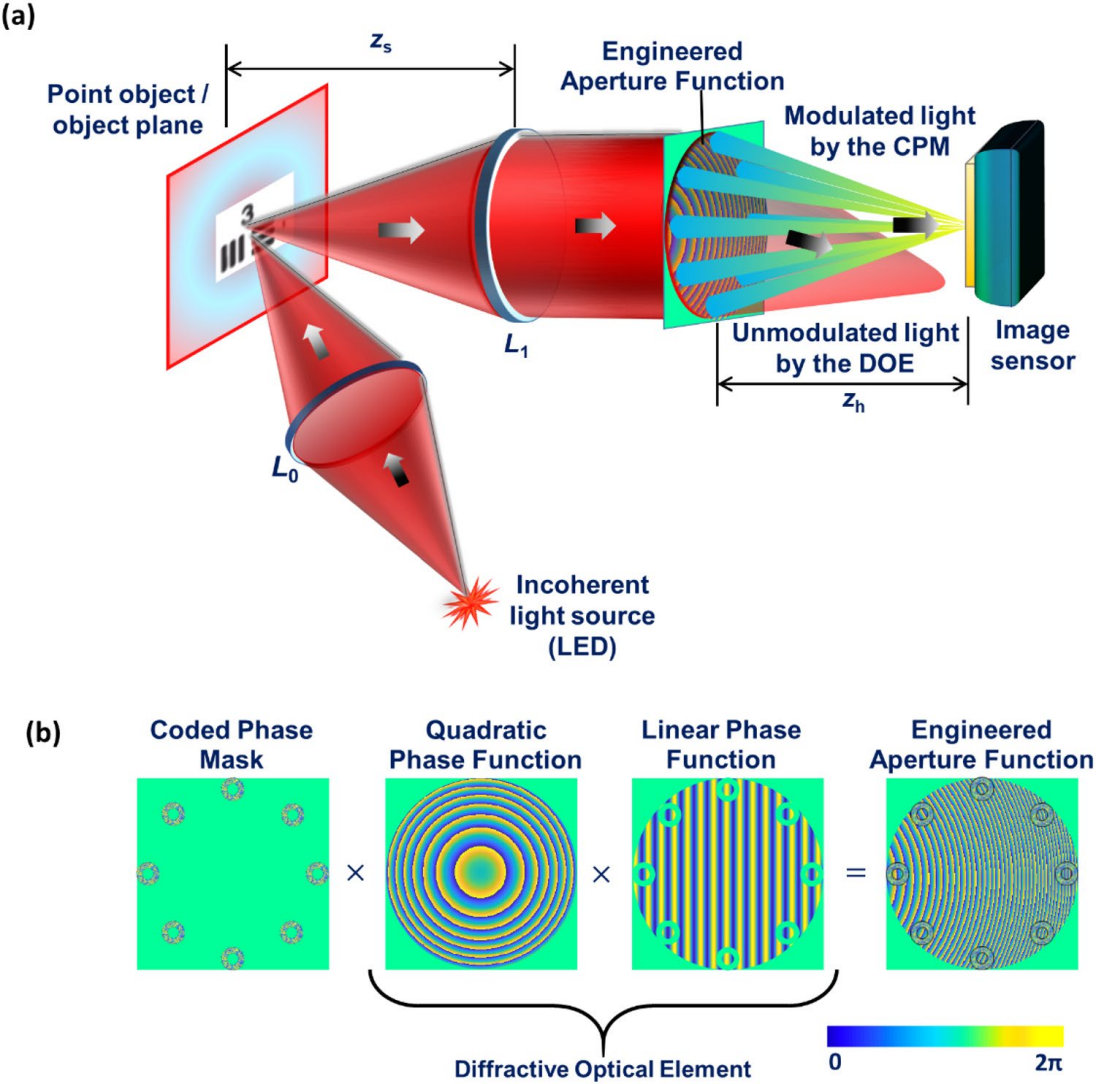
(**a**) A table-top telescopic M-PAIS model and (**b**) formation of the phase aperture. CPM—coded phase mask; *L*_0_ and *L*_1_—refractive lens; LED—light emitting diode and DOE—diffractive optical element.

A simplified tabletop M-PAIS model is shown in Fig. 2a. An incoherent light source illuminates in a critical illumination condition, either a point [not shown in Fig. 2a], or two-dimensional (2D) object, in a reflection mode via lens *L*_0_. The point object imitates a guidestar in the calibration stage of the system for recording point spread holograms (PSHs) which are later used for reconstructing images. The diffracted light from the point object is collimated by a refractive lens *L*_1_ to satisfy far-field imaging conditions. Thus, the object located at the front focal plane of lens *L*_1_ can be considered as an object placed at infinite distance from the imaging system. The collimated light is projected on an aperture function consisting of three components shown in Fig. 2b: a pseudorandom CPM in the shape of eight ring-shaped subapertures placed at equal angular positions, a quadratic phase function with focal length *z*_*h*_ and a linear phase function. The quadratic and the linear phase, denoted together as diffractive optical element (DOE), are designed to deviate unmodulated light by the CPM away from the sensor plane. The collimated light falling on subapertures is modulated by the CPM, and projected as an ensemble of dots on the sensor plane at a distance of *z*_*h*_ from the aperture plane, due to the above-mentioned quadratic phase of focal length *z*_*h*_.

**Figure 3:**
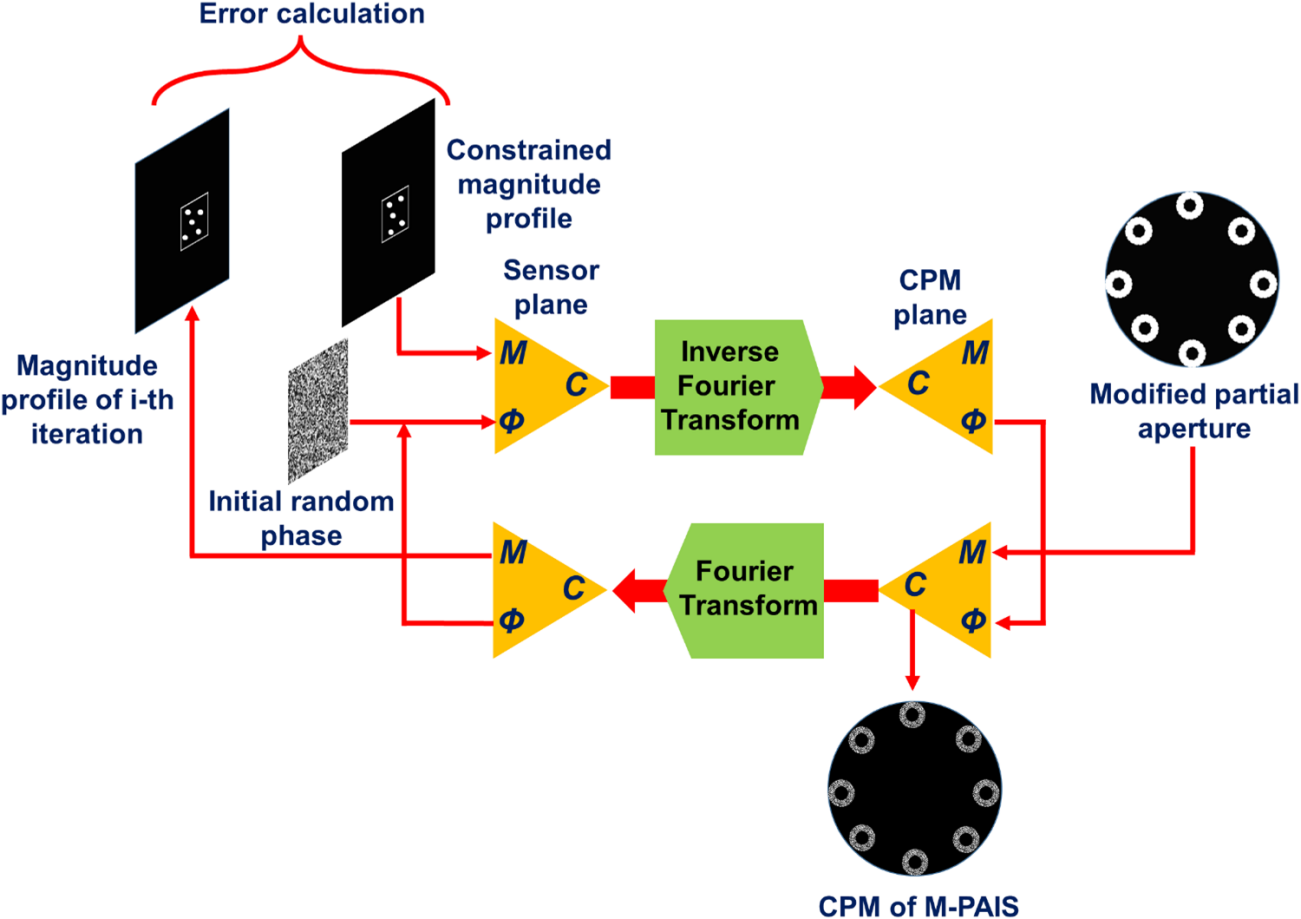
Gerchberg–Saxton algorithm for M-PAIS CPM synthesis.

Two independent pseudorandom CPMs, for the two camera shots, are synthesized using Gerchberg-Saxton algorithm (GSA)^29^. The GSA, shown in Fig. 3, is an iterative algorithm for computing a CPM constrained to produce randomly distributed intensity dots on a limited area of the sensor plane. The GSA starts from the sensor plane with a random phase function and unit magnitude dots randomly distributed in a predefined area. This initial function is transformed by an inverse Fourier transform to the CPM side, in which a desired partial aperture replaces the magnitude, whereas the phase distribution is kept the same for the next iteration. The modified function is Fourier transformed from the CPM plane to the sensor plane, where the mean square error (MSE) between the desired and the obtained magnitude profiles is calculated. After a few hundred iterations, when the MSE is smaller than a predefined threshold, the GSA is halted and the obtained CPM is ready for the experiment.

The following mathematical formulation is related to the optical configuration shown in Fig. 2a. A point object emitting intensity *I*_*s*_ is placed at the location $\,\left( {\overline{r}_{s} , - z_{s} } \right) = \left( {x_{s} ,{\text{y}}_{s} , - z_{s} } \right)$, a distance *z*_*s*_ from lens *L*_1_. We assume that the phase aperture is displayed on a spatial light modulator (SLM), and to make the analysis simpler, we neglect the distance between the SLM and the lens *L*_1_. The complex amplitude just before the SLM is given as $\sqrt {I_{s} } C_{0} L( - \overline{r}_{s} {/}z_{s} )Q(1{/}z_{s} )Q\left( { - 1{/}f_{1} } \right),$ where *C*_0_ is a constant. *L*(·) represents linear phase function given by $L(\overline{a}{/}z) = \exp \left[ {i2\pi \left( {\lambda z} \right)^{ - 1} \left( {a_{x} x + a_{y} y} \right)} \right]$, *Q*(·) is a quadratic phase function given by $Q(b) = \exp \left[ {i\pi b\lambda^{ - 1} \left( {x^{2} + y^{2} } \right)} \right]$ and *f*_1_ is the focal length of the collimating lens *L*_1_. The DOE, deviating the light away from the sensor, and the complex aperture of equally spaced *M* ring-shaped pseudorandom subapertures multiplied by the quadratic phase, are all displayed on the SLM. However, at the sensor plane, only the light modulated by the CPM is recorded. The *k*-th CPM transparency obtained by the GSA is,

1 $$P_{k} \left( {\overline{r}} \right) = \sum\limits_{m = 1}^{M} {\exp \left[ {i\Phi_{k,m} \left( {\overline{r}} \right)} \right]\left( {{\text{Circ}}\left( {\frac{{\left| {\overline{r}} \right|}}{{r_{1} }}} \right) - {\text{Circ}}\left( {\frac{{\left| {\overline{r}} \right|}}{{r_{2} }}} \right)} \right) * \delta \left( {\overline{r} - \overline{r}_{m} } \right)} ,$$

where $*$ is the sign of convolution, *δ* is the Dirac delta function, $\Phi_{k,m} \left( {\overline{r}} \right)$ is the pseudorandom phase of the *m*-th subaperture, ${\text{Circ}}\left( {{{\left| {\overline{r}} \right|} \mathord{\left/ {\vphantom {{\left| {\overline{r}} \right|} {r_{1,2} }}} \right. \kern-\nulldelimiterspace} {r_{1,2} }}} \right) = 1\;{\text{for}}\;\left| {\overline{r}} \right| \le r_{1,2} \;$ and 0 otherwise. *r*_1_ and *r*_2_ are the outer and the inner radiuses of the *M*-th subaperture, respectively, centered around a grid of *M* points at the annular position. The *k*-th impulse response recorded at the sensor plane is,

2 $$\begin{aligned} & I_{IR,k} \left( {\overline{r}_{0} ;\overline{r}_{s} ,z_{s} } \right) = \left| {\sqrt {I_{s} } C_{0} Q\left( {\frac{1}{{z_{s} }}} \right)L\left( {\frac{{\overline{r}_{s} }}{{z_{s} }}} \right)Q\left( { - \frac{1}{{f_{1} }}} \right)} \right.P_{k} \left( {\overline{r}} \right)Q\left( { - \frac{1}{{z_{h} }}} \right)\left. {*Q\left( {\frac{1}{{z_{h} }}} \right)} \right|^{2} \\ & \quad \quad \quad \quad \quad \quad = \left| {\nu \left[ {\frac{1}{{\lambda z_{h} }}} \right]\mathcal{F}\left\{ {\sqrt {I_{s} } C_{0} L\left( {\frac{{\overline{r}_{s} }}{{z_{s} }}} \right)Q\left( {\frac{1}{{z_{1} }}} \right)P_{k} \left( {\overline{r}} \right)} \right\}} \right|^{2} , \\ \end{aligned}$$

where $\mathcal{F}\left\{ \cdot \right\}$ indicates two-dimensional Fourier transform, *z*_1_ = *z*_*s*_*f*_1_/(*f*_1_-*z*_*s*_) and *ν* is the scaling operator such that *ν*[α]*f*(*x*) = *f*(α*x*).

Because the impulse response of Eq. (2) is inherently real and positive, its autocorrelation yields unaccepted background noise in the reconstruction plane. Thus, a single impulse response is less than optimal to be used as the PSH, and the same conclusion is valid regarding a single object response to be used alone as the object hologram. Therefore, we use two camera shots with two independent CPMs in order to generate bipolar holograms, in which their autocorrelations yield a sharp peak with negligible background level. The bipolar PSH in case of two camera shots is given by,

3 $$H_{PSH} \left( {\overline{r}_{0} ;z_{s} } \right) = I_{IR,1} \left( {\overline{r}_{0} ;0,z_{s} } \right) - I_{IR,2} \left( {\overline{r}_{0} ;0,z_{s} } \right).$$

A 2D object placed at the same location of the point object is considered as a collection of *N* uncorrelated intensity points given as,

4 $$O\left( {\overline{r}_{s} } \right) = \sum\limits_{j}^{N} {c_{j} \delta \left( {\overline{r}_{s} - \overline{r}_{j} } \right)} .$$

The assumption of uncorrelation is valid as long as the object is illuminated by a spatially incoherent quasi-monochromatic light source, and hence any two point-responses are not mutually interfered. Each *c*_*j*_ in Eq. (4) is a real positive valued intensity of the *j-*th object point at the location $\overline{r}_{j}$. The *k*-th object intensity response on the sensor plane is the sum of the shifted and scaled impulse responses given by,

5 $$I_{OR,k} \left( {\overline{r}_{0} ;\overline{r}_{s} ,z_{s} } \right) = \sum\limits_{j}^{N} {c_{j} I_{IR,k} \left( {\overline{r}_{0} - \frac{{z_{h} }}{{z_{s} }}\overline{r}_{j} ;0,z_{s} } \right)} .$$

Based on Eq. (5), the intensity on the sensor plane is the convolution between the object hologram and the PSH of the system. Similar to the PSH derived in Eq. (3), the final object hologram is the difference between the two responses recorded by the two camera shots with the same two independent CPMs as follows,

6 $$\begin{aligned} H_{OH} \left( {\overline{r}_{0} ;z_{s} } \right) & = I_{OR,1} \left( {\overline{r}_{0} ;\overline{r}_{s} ,z_{s} } \right) - I_{OR,2} \left( {\overline{r}_{0} ;\overline{r}_{s} ,z_{s} } \right) \\ & = \sum\limits_{j}^{N} {c_{j} \left[ {I_{IR,1} \left( {\overline{r}_{0} - \frac{{z_{h} }}{{z_{s} }}\overline{r}_{j} ;0,z_{s} } \right) - I_{IR,2} \left( {\overline{r}_{0} - \frac{{z_{h} }}{{z_{s} }}\overline{r}_{j} ;0,z_{s} } \right)} \right]} \\ & = O\left( {\frac{{z_{s} }}{{z_{h} }}\overline{r}_{0} } \right) * H_{PSH} \left( {\overline{r}_{0} ;z_{s} } \right) = \sum\limits_{j}^{N} {c_{j} } H_{PSH} \left( {\overline{r}_{0} - \frac{{z_{h} }}{{z_{s} }}\overline{r}_{j} ;z_{s} } \right). \\ \end{aligned}$$

From this point on, it is assumed that *z*_*s*_ = *f*_1_ and hence, according to Eq. (2), the *k*-th impulse response is a magnitude square of the scaled Fourier transform of the *k*-th CPM.

The way to reconstruct the image of the object $O\left( {\overline{r}_{s} } \right)$ is to reconstruct the series of Delta functions given in Eq. (4). Such reconstruction can be achieved if the sum of Eq. (6) is correlated with a function related to *H*_*PSH*_ that can yield as sharpest as possible Delta-like functions. At this point, we follow the method of^27^ to yield the series of sharp Delta-like functions by nonlinear parametric correlation which its formula is as follows,

7 $$I_{{\text{Im}}} = \mathcal{F}^{ - 1} \left\{ {\left| {\widetilde{H}_{OH} } \right|^{o} } \right.\left| {\widetilde{H}_{PSH} } \right|^{p} \left. {\exp \left( {i\left[ {\arg \left\{ {\widetilde{H}_{OH} } \right\} - \arg \left\{ {\widetilde{H}_{PSH} } \right\}} \right]} \right)} \right\},$$

where the parameters *o* and *p* are real numbers called coefficients of the nonlinear correlation^27^ for object and point object, respectively. $\widetilde{H}_{OH}$ and $\widetilde{H}_{PSH} \;$ are the 2D Fourier transforms of *H*_*OH*_ and *H*_*PSH*_, respectively. By varying *o* and *p*, the optimal reconstruction with the highest SNR values can be found. The values of *o* and *p* vary between the inverse filter value (*o* or *p* =  − 1) to matched filter (*o* = *p* = 1) i.e. $- 1 \le o,p \le 1$. Theoretically, *o* and *p* that satisfy the equation *o* + *p* = 0 are supposed to provide the best reconstruction results, but practically, in a noisy optical environment, this equation usually does not provide the sharpest reconstructions^28^. To obtain the best images, *o* and *p* are evaluated by varying in steps of 0.04 between $- 1$ to 1 and comparing SNR of the reconstructed images. Note that the special case of *o* = 1 and *p* = 0, applied in the previous works of PAIS^19,30^, is a linear correlation with a phase-only filter in the spectral domain. We also note that the option of the single camera shot^27^ can be realized by replacing $\widetilde{H}_{OH}$ and $\widetilde{H}_{PSH} \;$ in Eq. (7) with the Fourier transforms of $I_{OR,k}$ and $\,I_{IR,k}$, respectively, and this option is tested and compared in the following experiments.

The reasons for the superiority of one imaging method over another should be searched in the shape of the modulation transfer functions (MTF). The MTF operates as the filter in linear systems and as much as the MTF filters out less frequency components of the object spectrum, the image is more similar to the original object. In the present case, the imaging system defined by Eq. (7) is not linear and therefore to calculate the effective MTF of M-PAIS, we make a linearization of the analysis which is actually calculating the MTF of the closest linear system to the nonlinear M-PAIS. We start the calculation by rewriting Eq. (7) as the nonlinear cross-correlation between *H*_*OH*_ and *H*_*PSH*_ as the following,

8 $$\begin{aligned} I_{{\text{Im}}} & = \mathcal{F}^{ - 1} \left\{ {\left| {\widetilde{H}_{OH} } \right|^{o} \exp \left( {i \cdot \arg \left\{ {\widetilde{H}_{OH} } \right\}} \right)} \right\} \otimes \mathcal{F}^{ - 1} \left\{ {\left| {\widetilde{H}_{PSH} } \right|^{p} \exp \left( {i \cdot \arg \left\{ {\widetilde{H}_{PSH} } \right\}} \right)} \right\} \\ & = \mathcal{F}^{ - 1} \left\{ {\left| {\mathcal{F}\left\{ {O * H_{PSH} } \right\}} \right|^{o} \exp \left( {i \cdot \arg \left\{ {\mathcal{F}\left\{ {O * H_{PSH} } \right\}} \right\}} \right)} \right\} \otimes \mathcal{F}^{ - 1} \left\{ {\left| {\widetilde{H}_{PSH} } \right|^{p} \exp \left( {i \cdot \arg \left\{ {\widetilde{H}_{PSH} } \right\}} \right)} \right\} \\ & = \mathcal{F}^{ - 1} \left\{ {\left| {\widetilde{O}\cdot\widetilde{ H}_{PSH} } \right|^{o} \exp \left( {i \cdot \arg \left\{ {\widetilde{O}\cdot\widetilde{H}_{PSH} } \right\}} \right)} \right\} \otimes \mathcal{F}^{ - 1} \left\{ {\left| {\widetilde{H}_{PSH} } \right|^{p} \exp \left( {i \cdot \arg \left\{ {\widetilde{H}_{PSH} } \right\}} \right)} \right\}. \\ \end{aligned}$$

At this point comes the approximation of the nonlinear to the closest linear cross-correlation,

9 $$\begin{aligned} I_{{\text{Im}}} & \cong \mathcal{F}^{ - 1} \left\{ {\left| {\tilde{O}} \right|\exp \left( {i \cdot \arg \left\{ {\tilde{O}} \right\}} \right)\left| {\widetilde{H}_{PSH} } \right|^{o} \exp \left( {i \cdot \arg \left\{ {\widetilde{H}_{PSH} } \right\}} \right)} \right\} \otimes \mathcal{F}^{ - 1} \left\{ {\left| {\widetilde{H}_{PSH} } \right|^{p} \exp \left( {i \cdot \arg \left\{ {\widetilde{H}_{PSH} } \right\}} \right)} \right\} \\ & = O\left( {\overline{r}_{s} } \right) * \mathcal{F}^{ - 1} \left\{ {\left| {\widetilde{H}_{PSH} } \right|^{o} \exp \left( {i \cdot \arg \left\{ {\widetilde{H}_{PSH} } \right\}} \right)} \right\} \otimes \mathcal{F}^{ - 1} \left\{ {\left| {\widetilde{H}_{PSH} } \right|^{p} \exp \left( {i \cdot \arg \left\{ {\widetilde{H}_{PSH} } \right\}} \right)} \right\} \\ & = O\left( {\overline{r}_{s} } \right) * \mathcal{F}^{ - 1} \left\{ {\left| {\widetilde{H}_{PSH} } \right|^{o} \exp \left( {i \cdot \arg \left\{ {\widetilde{H}_{PSH} } \right\}} \right)\left| {\widetilde{H}_{PSH} } \right|^{p} \exp \left( { - i \cdot \arg \left\{ {\widetilde{H}_{PSH} } \right\}} \right)} \right\} \\ & = O\left( {\overline{r}_{s} } \right) * \mathcal{F}^{ - 1} \left\{ {\left| {\widetilde{H}_{PSH} } \right|^{o + p} } \right\} = O\left( {\overline{r}_{s} } \right) * \mathcal{F}^{ - 1} \left\{ {\left| {\mathcal{F}\left\{ {I_{IR,1} - I_{IR,2} } \right\}} \right|^{o + p} } \right\}. \\ \end{aligned}$$

Based on Eq. (9), it is clear that the MTF is equal to $\;\left| {\mathcal{F}\left\{ {I_{IR,1} - I_{IR,2} } \right\}} \right|^{o + p}$. Since each impulse response *I*_*IR,*1_ and *I*_*IR,*2_ is obtained as the magnitude square of a scaled Fourier transform of each of the corresponding CPM, the approximated MTF is,

10 $$MTF = |CPM_{1}^{\iota } \otimes CPM_{1}^{\iota } - CPM_{2}^{\iota } \otimes CPM_{2}^{\iota } |^{o + p} ,$$

where $CPM_{1,2}^{\iota }$ are the two CPMs in coordinates of spatial frequency. The conclusion from Eq. (10) is that the cutoff frequency of the system is *D/*(*λz*_*h*_), where *D* is the distance between two farthest points of two opposite positioned subapertures. This cutoff frequency is the same as of direct imaging system with the same aperture, but as is shown in the experiment section the shape of the MTFs of the two systems is different. The performance of different imaging systems with various parameters and conditions are compared next.

## Experiments

**Figure 4:**
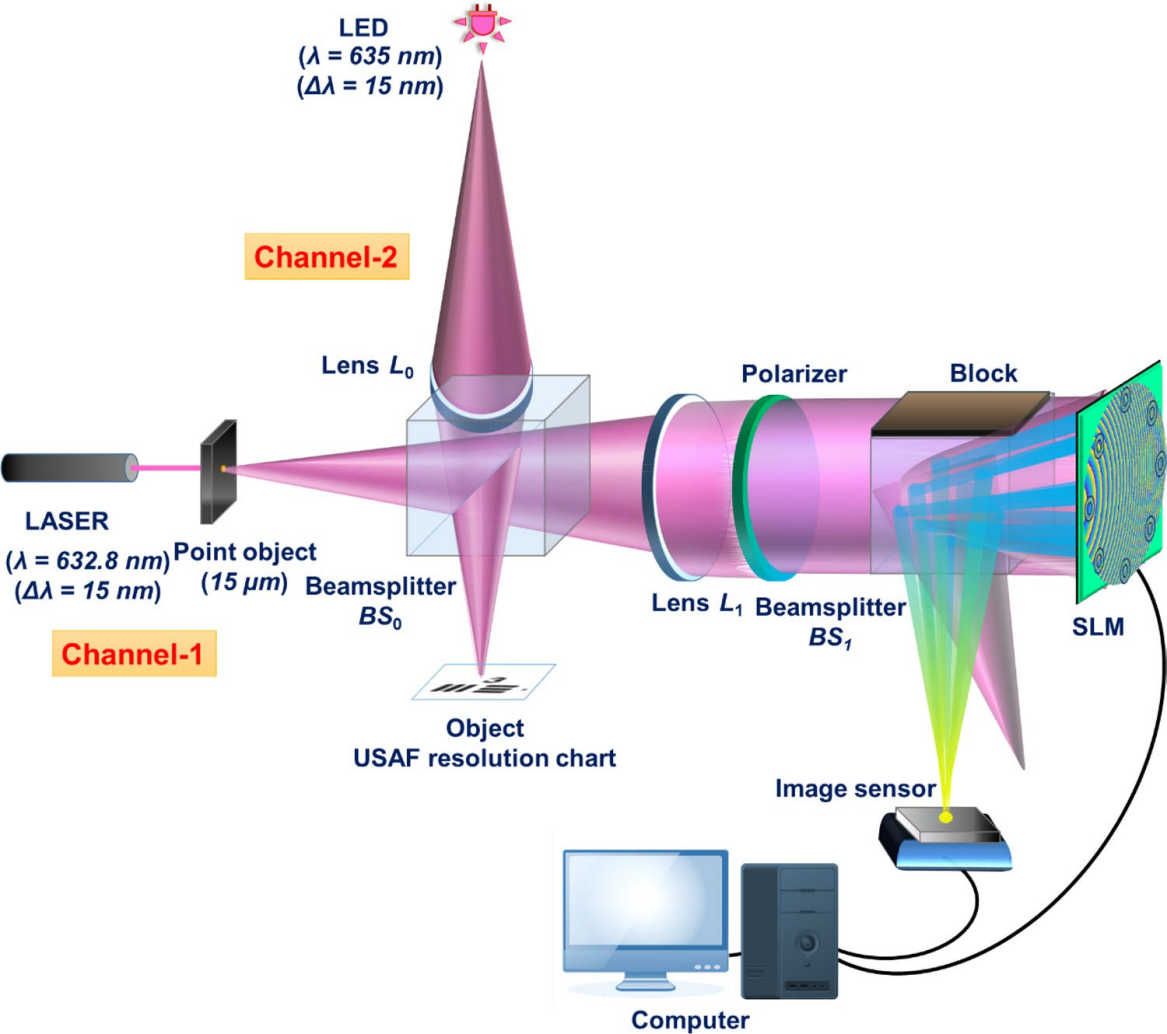
Experimental setup of M-PAIS. *BS*_0_ and *BS*_1_—beam splitters; *L*_0_ and *L*_1_—refractive lenses; LED—light emitting diode; SLM—spatial light modulator; USAF—United States Air Force.

The experimental setup of M-PAIS is shown in Fig. 4. The configuration has two illumination channels that have never been used simultaneously in this experiment. In the first channel, a HeNe laser (AEROTECH *λ* = 632.8 nm, max. output power 25 mW) illuminates a pinhole of 15 μm diameter used as the point object, whereas in the second channel, a LED (Thorlabs LED635L, 170 mW, *λ*_c_ = 635 nm, and Δ*λ* = 15 nm) illuminates an object via an objective lens *L*_0_. The laser is used only in the channel of the pinhole because it is more intense than the LED. The coherent nature of the laser is irrelevant in this system because the pinhole dictates the degree of the spatial coherence. In the object channel, according to the theory of the previous section, the reflected light from the object should be spatially incoherent, and hence the object is critically illuminated by the LED, where the slight difference between the wavelengths of the two sources is neglected. The third group, first element of a USAF resolution chart (Positive U.S. Air Force MIL-STD-150A standard of 1951 Test Target, 3" × 3" Thorlabs) is used as a reflective object for the entire experiments. In the first stage of the experiment, the point response of each CPM is generated by illuminating the pinhole by the laser and recording the intensity response by a CMOS camera (2048 × 2048 pixels, 6.5 μm pixel pitch, monochrome, Hamamatsu ORCA-Flash4.0 V2 Digital CMOS). The light diffracted from the pinhole passes through a beamsplitter *BS*_0_, and is collimated by the lens *L*_1_ with a diameter of 2.5 cm and a focal length of 20 cm located at a distance of 20 cm from the point object. A polarizer with polarization axis along the active axis of the SLM (1920 × 1080 pixels, 8 μm pixel pitch, phase-only modulation, Holoeye PLUTO) is mounted beyond lens *L*_1_. On the SLM, an aperture function is displayed, containing a CPM with 8 subapertures synthesized by GSA and imbedded into the DOE. The subapertures of the CPM are tested in three different sizes to investigate the influence of the size on the results. Additionally, for comparison purposes, we test two shapes for the subapertures, one is a ring and the other is a circular, both have the same area. The outer radii of the ring-shaped subapertures are *r*_11_ = 1, *r*_21_ = 0.75 and *r*_31_ = 0.5 mm; inner radii are *r*_12_ = 0.6, *r*_22_ = 0.45 and *r*_32_ = 0.3 mm. The radii of the equivalent circular aperture are *r*_1_ = 0.8, *r*_2_ = 0.6, and *r*_3_ = 0.4 mm. Thus, the areas of the subapertures are 2.01, 1.13 and 0.50 mm^2^. The *NA* of the system is 0.0216 with respect to full aperture of the SLM with a diameter of 8.64 mm. Finally, intensity patterns corresponding to the various CPMs were recorded by the camera, mounted at a distance of 25 cm from the SLM. After recording the two point-responses, the two object-responses were recorded.

## Experimental results

As described in the methodology section the CPMs are computed by the use of the modified GSA. To obtain optimal CPMs with minimum error, the MSE between the desired and the obtained dot patterns at the sensor plane, is calculated in each iteration as follows,

11 $$MSE = \frac{1}{MN}\sum\limits_{m = 1}^{M} {\sum\limits_{n = 1}^{N} {\left| {I_{o} \left( {m,n} \right) - \gamma I_{i} \left( {m,n} \right)} \right|^{2} } } ,$$

where $I_{o}$ is the desired dots and $I_{i}$ is the pattern obtained after each iteration^31^. The parameter $\gamma$ in Eq. (11) is,

12 $$\gamma = \frac{{\sum\limits_{m = 1}^{M} {\sum\limits_{n = 1}^{N} {I_{o} \left( {m,n} \right)I_{i} \left( {m,n} \right)} } }}{{\sum\limits_{m = 1}^{M} {\sum\limits_{n = 1}^{N} {|I_{i} \left( {m,n} \right)|^{2} } } }}$$

**Figure 5:**
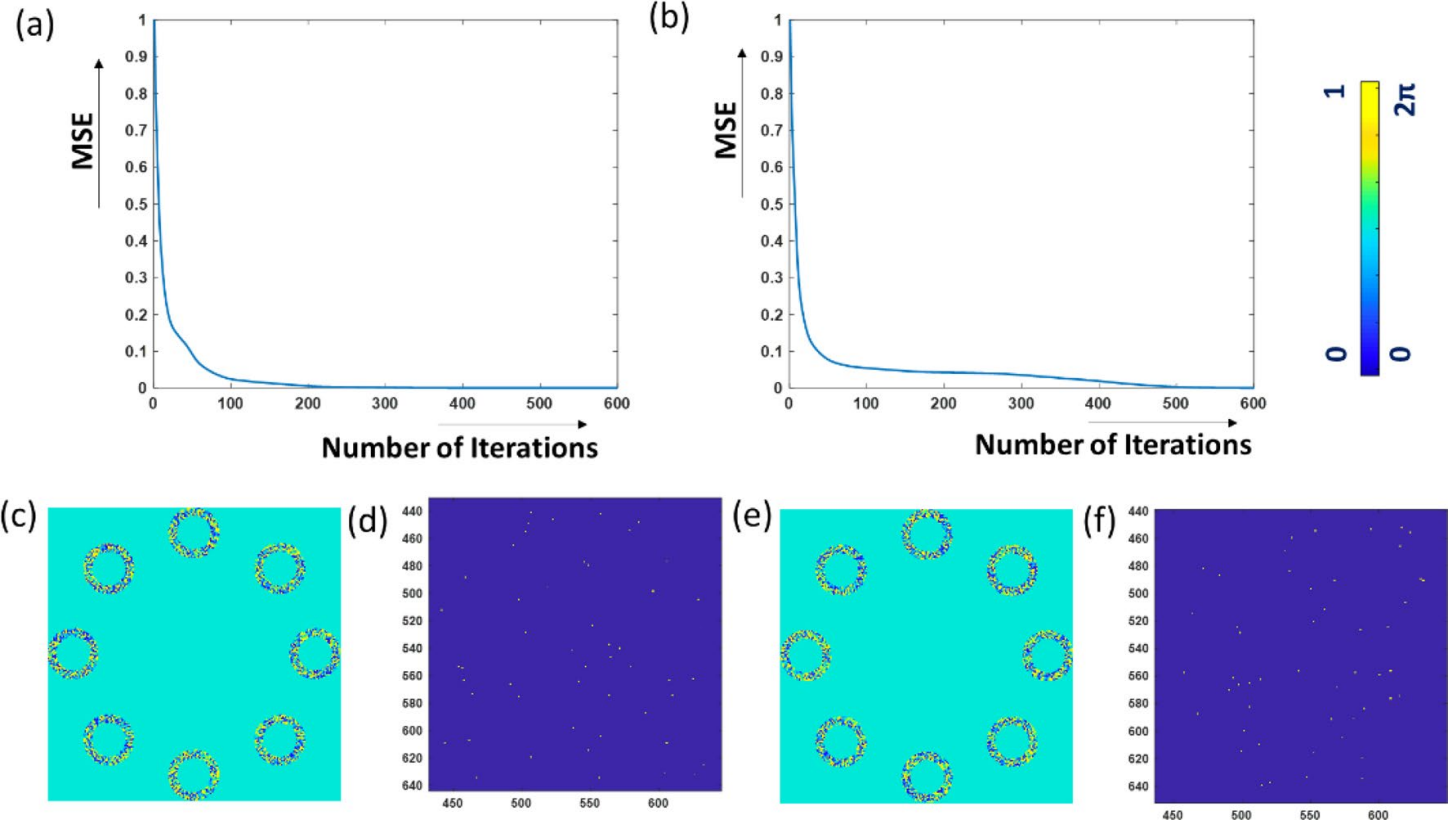
(**a**,**b**) MSE plots obtained during the synthesis of two independent CPMs; The obtained (**c**) CPM_1_ and (**e**) CPM_2_ by GSA; the desired (**d**) sparse intensity pattern of CPM_1_ and (**f**) the dotted intensity pattern of CPM_2_.

The MSE plots along 600 iterations for the two CPMs are shown in Fig. 5a and 5b. Their respective CPMs are shown in Fig. 5c and 5e. Figure 5d and 5f present the desired random dots intensity pattern with 50 random dots at the central area of 200 × 200 pixels on the sensor plane. Apparently, for the case of M-PAIS, beyond 600 iterations the MSE is close enough asymptotically to a minimum saturated value.

To compare the various imaging methods, four different experiments are carried out, each of which is tested for the subaperture of eight rings (M-PAIS) and eight complete circles (PAIS). In the first examined method the PSH is made of sparse dots obtained from two camera shots with two independent CPMs. In the second experiment, we use only one camera shot with a single CPM. In the third test, a continuous point response instead of the sparse dot response is used as the PSH. Finally, in the fourth experiment, direct images of the target are recorded by a diffractive lens displayed on the area of the subapertures, and the CPMs are removed. As mentioned above, the entire experiments are conducted for three sizes of subapertures.

**Figure 6:**
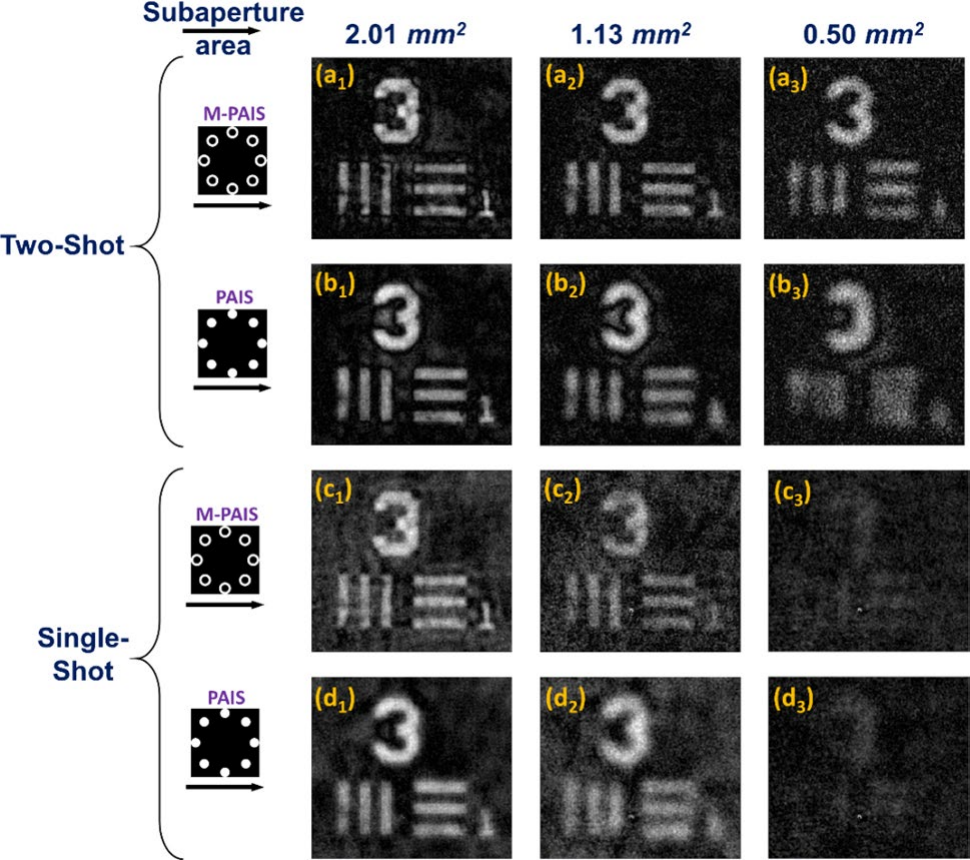
NLR results obtained from bipolar sparse holograms for, (**a**_**1**_**–a**_**3**_**)** M-PAIS with NLR coefficients *o* = 0.32, 0.56, 0.32 and *p* = 0.26, 0.24, 0.6; (**b**_**1**_**–b**_**3**_) PAIS with NLR coefficients *o* = 0.36, 0.2, 0.48 and *p* = 0.36,0.56, 0.32; NLR results of single camera shot of sparse hologram for (**c**_**1**_**–c**_**3**_) M-PAIS with NLR coefficients *o* = 0.24, 0.32, 0.44 and *p* = 0.48, 0.52, 0.28; (**d**_**1**_–**d**_**3**_) PAIS with NLR coefficients *o* = 0.28, 0.2, 0.28 and *p* = 0.4, 0.48, 0.44; subscript numbers represent images obtained from subaperture area of 2.01, 1.13 and 0.50 mm^2^.

**Figure 7:**
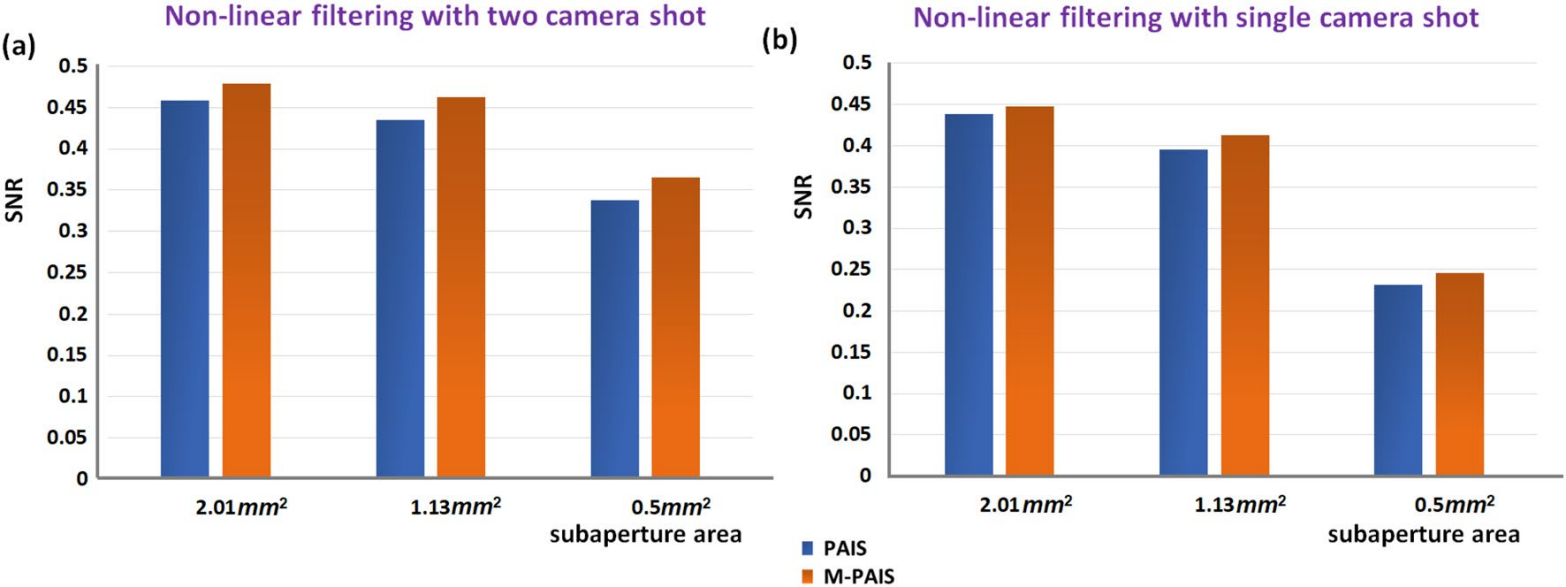
SNR of best NLR results obtained for M-PAIS and PAIS of (**a**) bipolar sparse holograms created from two-shot and (**b**) single hologram created from a single camera shot.

For PAIS and M-PAIS, the optimal values of parameters *o* and *p* of the NLR in the sense of highest SNR are searched and the reconstruction results shown in Fig. 6 are computed according to Eq. (7) for cases of a single and double CPM. The SNR is calculated according to the definitions of Gonzalez et al.^32^ as the following,

13 $$SNR = 10 \cdot \log_{10} \left[ {\frac{{\sum\nolimits_{m = 1}^{M} {\sum\nolimits_{n = 1}^{N} {\left| {O_{DI} (m,n)} \right|^{2} } } }}{{\sum\nolimits_{m = 1}^{M} {\sum\nolimits_{n = 1}^{N} {\left| {O_{DI} (m,n) - I_{Im} (m,n)} \right|^{2} } } }}} \right]$$

where *O*_*DI*_ is the desired image obtained by direct imaging with the full aperture. *I*_*Im*_ is obtained by NLR with the parameters $- 1 \le o,p \le 1$. Best nonlinear reconstructions using bipolar hologram for M-PAIS and PAIS are shown in Figs. 6(a_1_–a_3_) and 6(b_1_–b_3_), while the optimal reconstructions with a single hologram are shown in Figs. 6(c_1_–c_3_) and 6(d_1_–d_3_. The optimal values of *o* and *p* for each case are mentioned in the figure caption of Fig. 6. Their respective SNR values are shown in Figs. 7a and 7b. These plots indicate the superiority of M-PAIS over PAIS. Unlike the previous study of PAIS^19^, where several independent reconstructed images were averaged to improve the quality of the images, in the case of nonlinear image reconstruction with chaotic sparse holograms there is no need for averaging. This is because the use of sparse dot response and NLR improve the SNR of the reconstructed images in comparison to the continues response and linear reconstruction performed in^19,25^.

**Figure 8:**
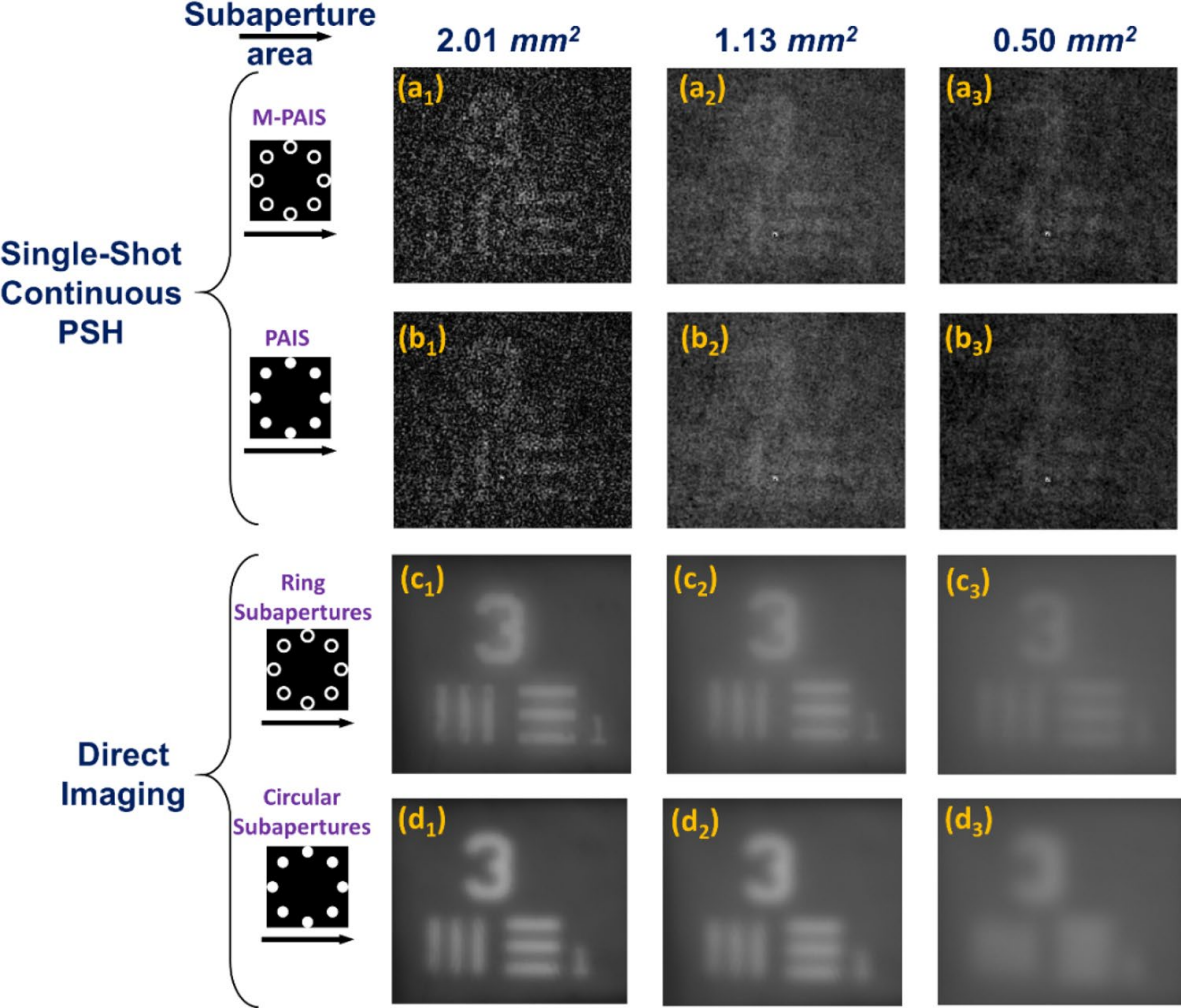
NLR results obtained by PSHs of continuous intensity profile, with a single-shot of (**a**_**1**_**–a**_**3**_) M-PAIS with NLR coefficients *o* = 0.32, 0.36, 0.4 and *p* = 0.28, 0.32, 0.32; (**b**_**1**_**–b**_**3**_) PAIS with NLR coefficients *o* = 0.32, 0.36, 0.32 and *p* = 0.24, 0.32, 0.40. Direct imaging through (**c**_**1**_**–c**_**3**_) ring and (**d**_**1**_**–d**_**3**_) circular subapertures; subscript numbers represent images obtained from subaperture area of 2.01, 1.13 and 0.50 mm^2^.

Figure 8(a_1_–a_3_) and 8(b_1_–b_3_) show the reconstructed images obtained for continuous point response on the sensor plane with a single camera shot and with NLR. Figure 8(c_1_–c_3_) and 8(d_1_–d_3_) are the direct images obtained without CPMs and where the light is focused on the camera by single quadratic phase function through 8 marginal rings in Figs. 8(c_1_-c_3_) and 8 circular subapertures in Figs. 8(d_1_-d_3_). Comparing the single-shot results of Figs. 6(c_1_-c_3_) and 6(d_1_-d_3_) with Figs. 8(a_1_–a_3_) and 8(b_1_–b_3_), reconstructions obtained from sparse holograms are always better than the results of continuous intensity holograms. This superiority is mainly due to the fact that the light power is concentrated in a relatively low number of dots instead of spreading all over the entire camera pixels. Hence, the signal of the dots is well above the noise level of the camera, making the SNR of the system higher than the case of the continuous PSH. Thus, for reflective objects, the sparse PSH is preferred over the continuous pattern. Between the two shapes of the subaperture the ring is preferred over the circle.

From Figs. 8(c_1_–c_3_) and 8(d_1_–d_3_), it is evident that direct imaging with ring-shaped subapertures has a better resolution than the circular subapertures with the same area. These results match with the results of sparse M-PAIS and sparse PAIS given in Fig. 6. When only PAIS and M-PAIS results with the same parameters are compared, M-PAIS resolves better than PAIS. The reason for this superiority can be understood from a comparative study of the MTFs. If PAIS, M-PAIS and direct imaging results are compared for the minimal subaperture area (0.50 mm^2^), the ring-shaped subaperture results with two shots are always better. Sparse M-PAIS and sparse PAIS are better than direct imaging in the sense of higher SNR, visibility and resolution.

**Figure 9:**
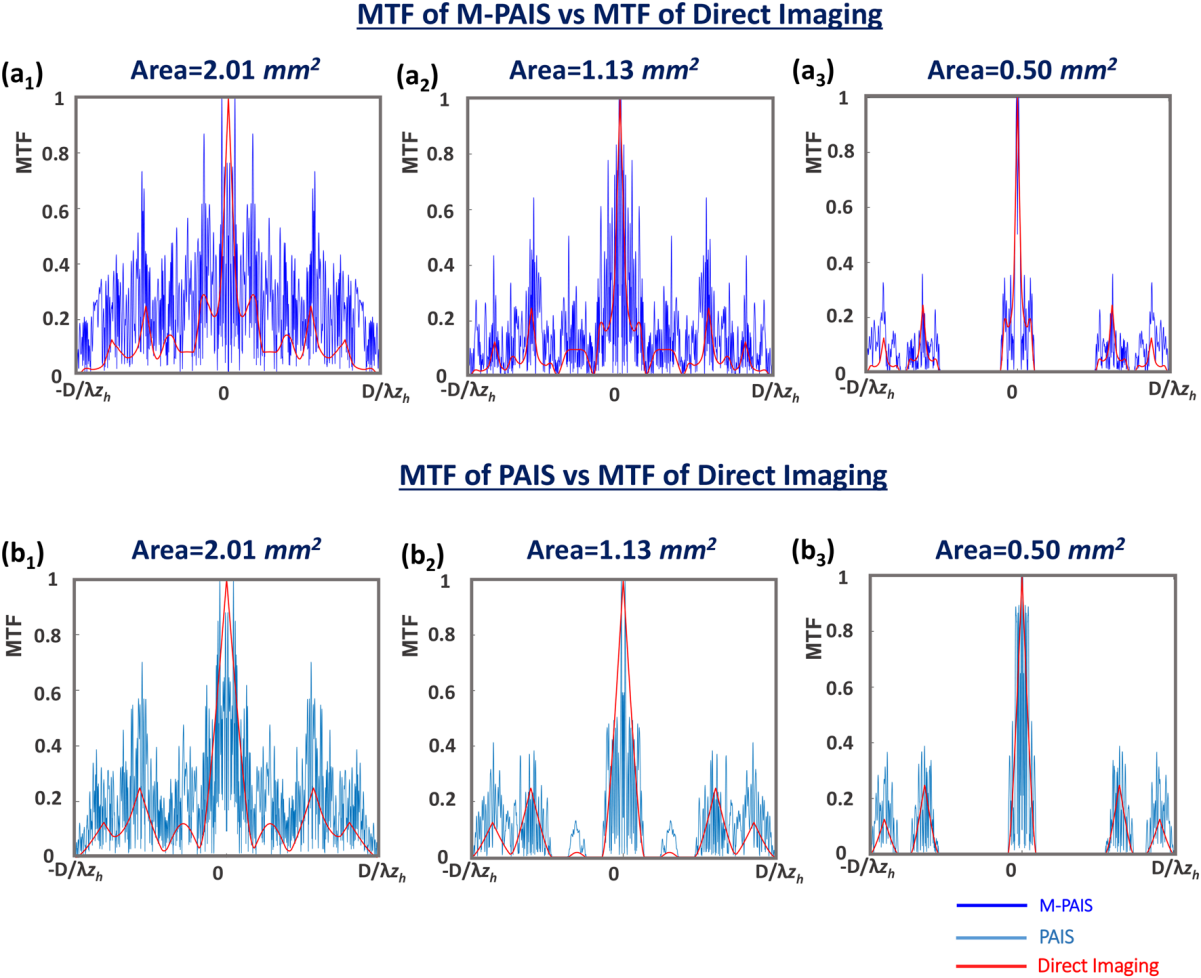
MTF cross-section plots for (**a**_**1**_**–a**_**3**_) M-PAIS and (**b**_**1**_**–b**_**3**_) PAIS in comparison to direct imaging for subaperture area of 2.01, 1.13 and 0.50 mm^2^.

The cross-sections of MTFs for various imaging systems are shown in Fig. 9. Comparing the various MTFs, the amplitude of the MTFs of M-PAIS and PAIS are more chaotic and on average has higher values than the smooth MTF of direct imaging. Comparing PAIS and M-PAIS in Figs. 9(a_1_–a_3_) and 9(b_1_–b_3_), the frequency coverage is larger and the uniformity is higher in the case of M-PAIS. These results of MTF comparison reflect the theoretical expectations that M-PAIS performance in general and resolution in particular will be superior over those of the other tested imaging methods. As mentioned in the methodology section, the annular subaperture has larger outer diameter than the circular subaperture for the same effective device area, and thus it provides better image resolution than PAIS. For example, comparing Figs. 9(a_2_) and 9(b_2_), one can see that M-PAIS with the area of 1.13 mm^2^ covers almost the entire spatial frequency range inside the bandwidth, whereas PAIS with the same area does not. Note that, the definition of MTF is valid for linear correlation, although the reconstruction methods of M-PAIS and PAIS used in this study are based on nonlinear correlations. Hence, the presented MTFs of the NLR are approximations according to Eq. (10).

**Figure 10:**
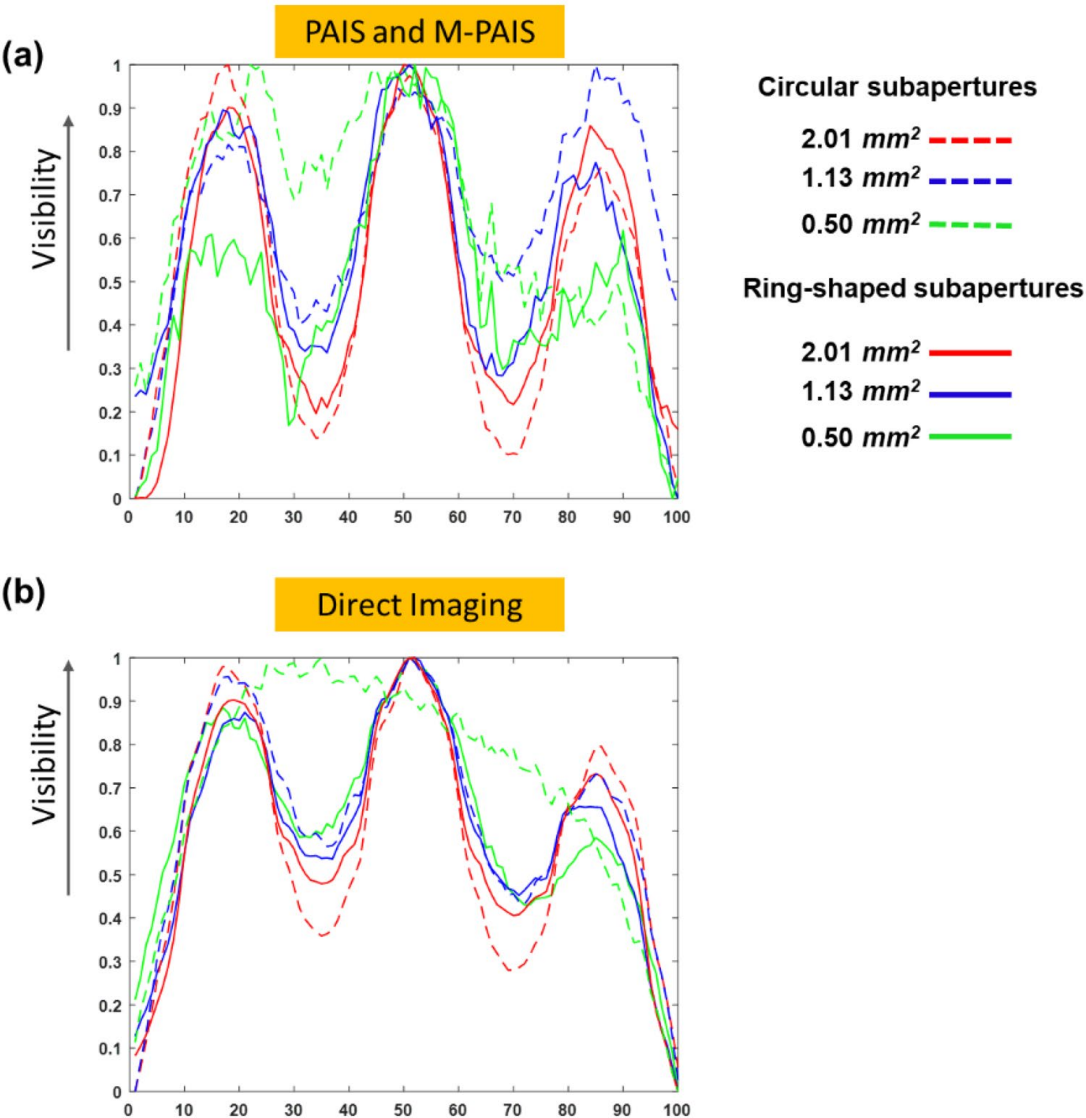
Visibility plots for (**a**) PAIS and M-PAIS and (**b**) direct imaging for circular subapertures and ring subapertures.

To further quantify the quality of the reconstructed images, normalized cross-sections of the reconstructed grating are presented in Fig. 10. According to Fig. 10, the visibility of M-PAIS and PAIS are better than of direct imaging even for the smallest subaperture area of 0.50 mm^2^, as is shown by the green curves. We conclude that optical telescopic systems like PAIS and M-PAIS potentially provide better images of reflective objects than the conventional direct imaging with a partial aperture^2^.

## Conclusion

In this article, a new configuration of a partial aperture imaging system termed M-PAIS is studied for reflective objects with a single and two camera shots. There are three main modifications in M-PAIS in comparison to the original PAIS^19,25^; 1. The PSH has been designed as an ensemble of sparse dots distributed randomly, instead of the continuous chaotic pattern. 2. M-PAIS is reconstructed by the nonlinear correlation in which its parameters are selected by an optimization process. 3. In M-PAIS we test sub-apertures with the shape of a ring instead of the open disk as previously. It turns out that each of these modifications and all of them together improve the reconstructed images in the sense of SNR, contrast and resolution.

The results obtained from M-PAIS, PAIS and direct imaging were compared under the same area value of the partial apertures. M-PAIS results were found to be better than PAIS and direct imaging primarily due to improvement in spatial frequency coverage. Thus, the M-PAIS system provides significant-resolution enhancement than its counterparts. The concept of PAIS and M-PAIS is investigated with reflective object to imitate the conditions of imaging passive non-self-luminous objects. The above mentioned three modifications enable the M-PAIS to image the reflective objects with better qualities (SNR and visibility) than the compared methods.

The comparison between the two shapes of the subapertures indicates that the area size of the subaperture is not the only important parameter of the subaperture, and its shape also plays an important role in the reconstruction quality. Moreover, we conclude that as much as the sparsity of the PSH in the sensor domain is important, the sparsity of apertures in the CPM domain is also significant, although from different reasons.

Another aspect studied herein is the number of camera shots. Instead, three camera shots as previously^19,25^, two, or even a single, camera shots are tested. The reduction in the number of shots improves the time resolution of the partial aperture systems. Although two camera shots are superior, the NLR enables the use of a single shot. Thus, the new modifications open new possibilities to image general astronomical objects by M-PAIS with comparable qualities of the full aperture imaging systems. Another application that could benefit from this study is the endoscope with the annular aperture proposed recently in^30^. The method enables SNR enhancement which is particularly useful for objects with dim light. By using the proposed method, a lot of optical hardware weight and a long exposure time can be saved. For future work, it might be interesting to investigate the concept of the synthetic aperture with only two sub-apertures of M-PAIS moving between all the eight positions of the current M-PAIS. It is interesting because such systems might provide the same quality of images by much less system weight albeit with longer acquisition time.
